# A putative cytotoxic serine protease from *Salmonella typhimurium* UcB5 recovered from undercooked burger

**DOI:** 10.1038/s41598-023-29847-8

**Published:** 2023-03-09

**Authors:** Essam Kotb, Baher A. El-Nogoumy, Haifa A. Alqahtani, Asmaa A. Ahmed, Hussah A. Al-shwyeh, Sakina M. Algarudi, Hanan Almahasheer

**Affiliations:** 1 Department of Biology, College of Science, Imam Abdulrahman Bin Faisal University (IAU), P.O. Box 1982, Dammam, 31441 Saudi Arabia; 2 Basic & Applied Scientific Research Center, Imam Abdulrahman Bin Faisal University, P.O. Box 1982, Dammam, 31441 Saudi Arabia; 3 Department of Botany and Microbiology, Faculty of Science, Kafrelsheikh University, Kafrelsheikh, 33516 Egypt; 4 Department of Statistics, Faculty of Commerce, Al-Azhar University (Girls’ Branch), P.O. Box 11751, Cairo, Egypt

**Keywords:** Bacterial physiology, Microbiology

## Abstract

A putative virulence exoprotease designated as UcB5 was successfully purified from the bacterium *Salmonella typhimurium* to the electrophoretic homogeneity with 13.2-fold and 17.1% recovery by hydrophobic, ion-exchange, and gel permeation chromatography using Phenyl-Sepharose 6FF, DEAE-Sepharose CL-6B, and Sephadex G-75, respectively. By applying SDS-PAGE, the molecular weight was confirmed at 35 kDa. The optimal temperature, pH, and isoelectric point were 35 °C, 8.0, 5.6 ± 0.2, respectively. UcB5 was found to have a broad substrate specificity against almost all the tested chromogenic substrates with maximal affinity against N-Succ-Ala-Ala-Pro-Phe-pNA achieving *K*_m_ of 0.16 mM, *K*_cat_/*K*_m_ of 3.01 × 10^5^ S^−1^ M^−1^, and amidolytic activity of 28.9 µmol min^−1^ L^−1^. It was drastically inhibited by TLCK, PMSF, SBTI, and aprotinin while, DTT, β-mercaptoethanol, 2,2′-bipyridine, *o*-phenanthroline, EDTA, and EGTA had no effect, which suggested a serine protease-type. Also, it has shown a broad substrate specificity against a broad range of natural proteins including serum proteins. A cytotoxicity and electron microscopy study revealed that UcB5 could cause subcellular proteolysis that finally led to liver necrosis. For this, future research should focus on using a combination of external antiproteases and antimicrobial agents for the treatment of microbial diseases instead of using drugs alone.

## Introduction

Toxins and enzymes operate as key virulence agents in microbial pathogenesis inside the diseased hosts^1,2^. Bacterial toxins are fully characterized and their role in the pathogenesis process is well-studied, whereas the role of microbial proteases in the pathogenesis of animals and plants is not well-studied. This could be a result of the complexity of enzymes and lack of selectivity in comparison to toxins^3^. Proteases have been identified a long time ago in the cellular extracts of many pathogenic bacteria^4^. It may come as a surprise to learn that the majority of them were unidentified for a long time and were only thoroughly defined recently. Microbes produce many types of proteases categorized as serine, metallo, aspartic, and cysteine types. Some of them are specifically inhibited by plasma protease inhibitors known as serpins, while most of them are resistant or inactivate human plasma antiproteases. As a result, once inside, these will accelerate the processing of disease and host impairment^5^.

Their involvement in virulence has been linked to a variety of methods. To begin the invasion process or digest host proteins to access peptidic nutrients, they first proteolyze and destroy host tissue. For instance, by interfering with cell signaling proteins or proteolyzing proteins of the matrix components, HtrA protease facilitates the spread of diseases first. Second, they may activate subunit toxins (A-B toxins) by splitting the active moiety (A subunit) from the binding moiety (B subunit). Third, some of them such as Clp and Lon proteases act inside the cytosol directly by well-timed destruction of virulence controllers and indirectly by providing resistance to the interior antagonistic components such as superoxides and free radicals to stop the host immune effector components against the infecting bacterial pathogen^6^.

*S. enterica* with more than 2000 serovars can cause many different diseases. Serovar Enteritidis and Typhimurium are the most common cause of gastroenteritis for humans, whereas other serovars like *S. typhi* are the fundamental cause of fatal systemic diseases. Interestingly, serovar Typhimurium mutants missing clpP or clpX proteases are found to be non-virulent strains indicating the significance of the ClpXP protease in salmonellosis^7^. Besides*,* serovar mutants lacking Lon protease are highly sensitive to H_2_O_2_ and acidity, therefore incapable to persist inside macrophages and proliferation in distal parts of the body to initiate diseases^8^. It was found that peptidase N of serovar Typhimurium is the primary aminopeptidase inside cytosols with a wide range of substrate activity^9^.

As of right now, infections caused by the serovars of *Salmonella* remain dangerous, especially in low- and middle-income countries where they can be consumed along with many contaminated foods and result in local pathological conditions inside the alimentary tract, that may spread into a systemic infection^10^. Unfortunately, detailed characterization of *Salmonella* proteases is lacking, therefore, we primarily intended to monitor the proteolytic and hemolytic activities of *Salmonella* and *Shigella* isolates accompanying local food samples. The goal of this part of the research was extended to reveal the enzyme characteristics and the cellular alterations of mammalian cells due to the UcB5 protease produced by the most potent isolate, *S. typhimurium*.

## Materials and methods

### Materials

DTT, SBTI, and EAM were obtained from Merck (Beijing, China). Fibrin, fibrinogen, pNA, PMSF, DMSO, EGTA, PHMB, leupeptin, and pepstatin A were procured from Sigma-Aldrich (MO, USA). Chromogenic synthetic substrates such as D-Val-Leu-Arg-pNA (V6258), D-Val-Leu-Lys-pNA (V7127), D-Phe-Pip-Arg-pNA (P7027), and N-Succ-Ala-Ala-Pro-Phe-pNA (S7388) were also purchased from Sigma-Aldrich. HT29 human adenocarcinoma cell line was purchased from Merck (Darmstadt, Germany). Phenyl-Sepharose 6FF, DEAE-Sepharose CL-6B, Sephadex G-75, and marker proteins were purchased from Amersham Biosciences (Sweden). The rest of the chemicals were of analytical grade from resident providers.

### Bacterial isolation and characterization

The bacterial strain under study was originally recovered from an undercooked beef burger sample that has been purchased from a local traditional market. This strain has shown the uppermost proteolytic and hemolytic activity among a total of fourteen bacterial isolates recovered from diverse food samples including processed meats and dairy products. Differential isolation of these bacteria was carried out on *Salmonella-Shigella* (SS) agar (Himedia, India) plates supplemented with 1% skim milk at pH 7.0. Thence, dishes were incubated at 37 °C for 48 h. Cleared halos surrounding colonies are indicative of proteolytic activity. For hemolytic activity screening, SS agar supplemented with 10% citrated sheep blood was used. Halo zones surrounding bacterial colonies were indicative of hemolytic activity. Based on this screening program, isolate number five which has been isolated from an undercooked beef burger sample was selected and identified to the species level using *16SrDNA* gene fingerprint and run BLAST analysis on the GenBank database. Short-term bacterial cultures were preserved on nutrient agar at 4 °C, while long-term cultures were preserved at − 80 °C in 20% (v/v) glycerated broth.

### Enzyme production

Inocula of strain number UcB5 were regularly subcultured in LB broth consisting of (g/L) NaCl 5.0, yeast extract 5.0, and tryptone 10.0 with pH 7.0. Exactly, one percent (v/v) of 12 h old inoculum (~ 3 × 10^8^ cfu/mL), was transported into the fermentation broth at pH 7.0. The basal medium consisted of (w/v) 1% fructose, 0.5% NaCl, 0.5% peptone, 0.15% MgSO_4_·7H_2_O, 0.08% KH_2_PO_4_, 0.02% K_2_HPO_4_, 0.005% CuSO_4_ and 0.001% FeSO_4_. Incubation was done under a shaking speed of 150 revolutions per minute at 37 °C for 48 h in 250 mL Erlenmeyer flasks holding 50 mL broth.

### Protease assay and protein quantification

The proteolytic activity was assessed by mixing 1 mL of culture supernatant and 1 mL of 1% (w/v) azocasein solution dissolved in 0.2 M Tris–HCl buffer of pH 7.0. The enzyme–substrate reaction was allowed to proceed for 30 min at 37 °C and was ended through the addition of 2 mL of 10% (w/v) trichloroacetic acid solution. Thence, incubation for 60 min in a crushed ice bath. The amount of soluble degradation proteins (C) was measured (mg/mL) following this calculation; C (mg/mL) = 1.55 OD_280_ − 0.76 OD_260_. One unit (1 U) of proteolytic enzyme activity was equivalent to one microgram of released l-tyrosine per milliliter per minute of the reaction at the standard conditions of experimentations.

### Enzyme purification

The crude protease was purified under cooling at 4 °C through four stages comprising (NH_4_)_2_SO_4_ salt precipitation, Phenyl-Sepharose 6FF hydrophobic chromatography, DEAE-Sepharose CL-6B anion exchange chromatography, and Sephadex G-75 gel permeation chromatography. Initially, the culture broth was centrifuged at 7000×*g* for 10 min, then the addition of (NH_4_)_2_SO_4_ at 30–70% saturation was performed. Centrifugation at a speed of 10,000×*g* for 15 min to harvest the soluble proteins. Pelleted crude protease and other proteins were then resuspended at pH 7.8 in buffer A (20 mM Tris–HCl comprising 1.0 M )NH_4_)_2_SO_4_. After the elimination of the suspended matter and the dialysis step, the concentrated crude protease was loaded into a Phenyl Sepharose 6 FF column of 1.5 × 20 cm^2^ dimension. Thereafter, a linear ascent of 0.5–0.0 M (NH_4_)_2_SO_4_ in buffer A was applied for the elution of proteins. From this elution chromatogram, active fractions showing enzymatic activity were pooled and concentrated. Thence, loaded onto the next column comprising of DEAE-Sepharose CL-6B with 1.5 × 15 cm^2^ dimension. Elution was done at a 0.5 mL/min flow rate at pH 9.4 with 20 mM Tris–HCl buffer (buffer B). Active fractions displaying proteolytic activity were then concentrated, dialyzed with buffer B, and loaded into the next column comprising Sephadex G-75 FF with a 1.5 × 30 cm^2^ dimension. Elution was done by buffer A. Final protease-active fractions were lyophilized and SDS-PAGE was performed by the universal technique of Laemmli^11^ using 15% (w/v) separating gel and 5% (w/v) stacking gel.

### Effect of temperature on protease activity and stability

The temperature impact on enzyme activity was examined at 20–65 °C using 1% (w/v) azocasein in 0.2 M Tris–HCl buffer, pH 7.0. On the other hand, to study the thermal stability, a tested protease solution in 0.2 M Tris–HCl (pH 7.0) was left to stand at 20 to 65 °C for 60 min. By the end of incubation, the treatments were then cooled down for the determination of remaining protease activity at the typical settings of enzyme assay.

### Effect of pH on protease activity and stability and determination of pI

For studying the impact of pH on the proteolytic activity of UcB5, the reacting solutions were fixed to a wide range of pHs by three buffers. Citrate–phosphate buffer to achieve a pH range of 3.0 up to 6.0, Tris–HCl buffer for pHs in the range of 7.0 up to 8.0, and glycine–NaOH buffer to adjust pHs in the range of 9.0 up to 13.0. Azocasein at 1% (w/v) concentration was used and incubation of enzyme–substrate reactions was done at 35 °C.

For studying the pH stability of the pure enzyme, it was incubated for 60 min at 35 °C with various pHs in the range of 5.0 up to 13.0 using the prescribed buffers. The remaining enzymatic activity was assessed at pH 8.0. Furthermore, the isoelectric point of the tested protease was determined by overnight incubation of a concentrated preparation of the pure enzyme at pHs in the range of 3.0 up to 11.0 at 4 °C. Protein precipitation was done at the gravitational force of 10,000*×g* for 15 min. The protein pellets were quantified according to Bradford^12^ method. The isoelectric point was expressed as the pH degree where maximal protein precipitation has been done^13^.

### Amidolytic activity and kinetic parameters of the purified UcB5

These were determined colorimetrically against several chromogenic peptides as synthetic substrates such as P7021, V7127, S7388, and V6258. For experimentation, each well of a microplate was loaded with 5 µl of enzyme solution in 20 mM Tris–HCl buffer (pH 8.0), and 100 µl of a synthetic substrate at a specific concentration from 0.02 up to 0.15 mM. The reaction was done at 37 °C and terminated at time intervals by the addition of 1.4 ml of 0.15 M trichloroacetic acid. The amount of released pNA was estimated spectrophotometrically at *A*_405_ nm. One unit (1 U) of amidolytic activity of protease was calibrated to nmol of the chromogenic substrate degraded per min per mL due to the action of tested protease. The kinetic parameters were estimated from the Lineweaver–Burk plots depending on the initial rate of the enzymatic reaction.

### Effect of metal ions and protease inhibitors on UcB5 activity

To define the group to which the purified UcB5 belongs, the influence of various metallic ions and standard reagents upon its amidolytic activity was explored. In a microplate, these were mixed with 1.0 × 10^–4^ mg of the chromogenic substrate S7388 and 2.0 × 10^–3^ mg of the enzyme in 100 µl of 20 mM Tris–HCl (pH 8.0) buffer and incubated for 3 min at 37 °C. The released pNA was quantified by the spectrophotometric measurement at *A*_405_.

For metal ions experimentation, they were tested in parallel tests at the concentration of 5 mM. The applied concentrations of protease reagents varied according to the cited literature (see the results). The enzyme activity in the treatment devoid of reagents and cations was considered 100%.

### Substrate specificity

The substrate specificity of UcB5 against natural protein substrates was studied at 0.5% (w/v) concentration following the procedure of Peng et al.^14^. These include casein, elastin, hemoglobin, fibrin, gelatin, fibrinogen, collagen, mucin, IgG, and serum albumin. This was determined against several natural proteins. One unit (1 U) of proteolytic activity was calibrated as the quantity of enzyme that releases the comparable to 1 µmol of the amino acid tyrosine per milliliter per minute under the standard conditions of the assay.

### In vitro anticoagulant activity

The next experiments were approved by an appropriate institution. In addition to this, all methods were performed following the relevant guidelines and regulations including ARRIVE guidelines. The in vitro anticoagulant activity was examined as the increase in the coagulation period of human blood serum in the existence of 15 µg of UcB5 protease/ml^15^. Exactly, 100 µl of blood serum was vortexed with equivalent volumes of each thromboplastin and kaolin. After 2 min incubation at 37 °C in a water bath, exactly 100 µl of 0.3% (w/v) CaCl_2_ and 100 µl of the enzyme were added. The clotting time in the presence of the enzyme was then determined in comparison with blanks containing an equivalent amount of physiological saline instead of the purified enzyme.

### Cell-damaging activity

The in vitro cytotoxicity assay of the purified protease of *S. typhimurium* against HT29 human adenocarcinoma cell line (Merck, Darmstadt, Germany) was performed on a 96-well plate by incubation for 24 h. Per 1 × 10^5^ HT29 cells per milliliter, fifteen micrograms of the enzyme were used. By the end of incubation, the percent of cell death of HT29 was assessed by the standard MTT assay^16^. For negative blanks, physiological saline was used instead of the active protease preparations, while for blanks, a medium without cells was used. The idea of this assay is that the remaining surviving cells can convert the yellow tetrazolium MTT reagent into the purple formazan complex with a characteristic absorption at *A*_540_ nm, therefore indicating HT29 viability. The intensity of the purple color is in direct relation to the number of surviving cells after exposure to the UcB5.

Furthermore, an assay of cell-damaging activity against RBCs (hemolytic activity) due to the purified enzyme was done. This was achieved by vortexing identical volume sizes of 15 µg protease/mL and 4% (v/v) washed human RBCs suspended in 0.1 M borate buffer, pH 7.5. Incubation was done at 37 °C for 90 min, thereafter, the quantity of released hemoglobin was measured colorimetrically. For comparison, a complete hemolysis treatment was done by mixing RBCs suspension with 1% (v/v) triton X-100 solution.

Also, an in vivo screening of cell-damaging activity was done and the LD_50_ value was calculated. For this, BALB/c mice weighing 22–25 g were acclimatized to the laboratory conditions for one week and retained at relatively fixed nutritional and physical conditions. They were then divided into six groups of six per cage. The first group was represented as the universal blank group. Mice in this group were intraperitoneally inoculated with an equal volume of a heat-denatured enzyme preparation at a concentration of 60 µg/body weight. While the other five groups were intraperitoneally injected with the active protease preparation at various concentrations (60, 30, 15, 8, and 4 µg protease/body weight) in a total volume of 1 ml solution. Animals were then observed at time intervals throughout 48 h for the LD_50_ calculation according to the method of Karber. Livers of affected and blank mice were removed instantly after death and fixed in 5% (v/v) glutaraldehyde then 1% (w/v) OsO_4_ solution. Before dissection of a blank mouse, it was anaesthsized by the inhalant gas sevoflurane. Ultrathin sections of 70 nm were sliced by RMC ultramicrotome and loaded on standard-grade TEM support grids made-up of copper for examination under JEOL 1010 TEM.

### Statistical analysis

All the measurements and treatments were performed in triplicates unless otherwise stated. The statistical analysis was done by SPSS Statistics V24 software. The final readings were represented in the form of averages ± standard deviations.

### Ethics approval

The in vivo study was done under permission No. 5/2019EC from the Experimental Animals Care Ethics Committee of Kafrelsheikh University, Egypt. In addition to this, all methods were performed in accordance with the relevant guidelines and regulations including ARRIVE guidelines.

## Results

During a preliminary survey for *Salmonella* and/or *Shigella* isolates exerting both proteolytic and hemolytic activity, we isolated a total of fourteen isolates on SS agar supplemented with skim milk. These were originally obtained from thirty food samples collected from some local markets. The most potent producer among them was isolate number five which has been isolated from an undercooked beef burger sample. Therefore, it was designated as strain UcB5. The *16SrDNA* gene sequencing data and BLAST analysis indicated that strain UcB5 was *S. typhimurium* with 98.86% similar identity with an existing genus and species. The nucleotide sequence was listed in the GenBank Records Library under the accession number (MH340533.1).

### Relation of growth phase with enzyme productivity

**Figure 1:**
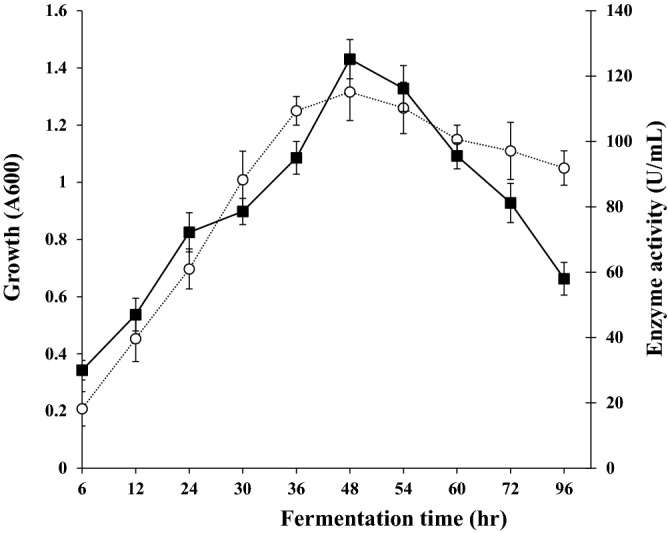
Relation of the protease production (-■-) and the growth phase (-○-) in a culture of *S. typhimurium* grown in a basal medium composed of (w/v) 1% fructose, 0.5% peptone, 0.5% NaCl, 0.15% MgSO_4_·7H_2_O, 0.08% KH_2_PO_4_, 0.02% K_2_HPO_4_, 0.005% CuSO_4_ and 0.001% FeSO_4_. Fermentation was done on a rotatory shaker at the speed of 150 rpm at 37 °C and an initial pH of 7.0 for 48 h. There is a highly positive correlation between bacterial growth and enzyme productivity since the correlation coefficient *r* = 0.886***. The two-tailed *P-*value is given as 0.00, this yields a very highly significant linear correlation.

The bacterial growth and the UcB5 protease production from the most potent strain were synchronized during the whole fermentation period (96 h). They reached the maximal levels at 48 h of incubation where the bacterial growth reached an optical density of 1.32 at wavelength 600 nm and the enzyme productivity reached 125.2 U/mL (Fig. 1).

### Purification of UcB5

**Figure 2:**
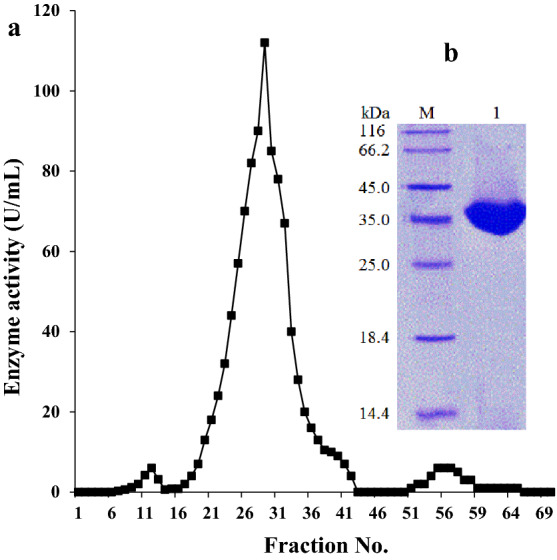
Elution profile of the UcB5 through Sephadex G-75 (**a**) and SDS-PAGE (**b**). Initially, the culture proteins were precipitated with ammonium sulfate at 30–70% concentrations then loaded into a Phenyl Sepharose 6FF column (1.5 × 20 cm^2^) followed by a DEAE-Sepharose CL-6B column (1.5 × 20 cm^2^). and a Sephadex G-75 FF column (2.5 × 100 cm^2^). Finally, SDS-PAGE analysis was done using 5% (w/v) stacking gel and 15% (w/v) separating gel. The gel is cropped with paint software and the previous version of gel is found in the Supplementary information file.

As briefed in Table 1, the UcB5 enzyme was efficiently purified to the electrophoretic homogeneity from *S. typhimurium* liquid cultures through various steps including hydrophobic, ion-exchange, and gel filtration chromatography by Phenyl-Sepharose 6FF, DEAE-Sepharose CL-6B, and Sephadex G-75, respectively. The final specific activity of the enzyme was increased to the fold of 13.2 × and the recovery of 17.1%. The electrophoretic homogeneity of the tested enzyme was reflected from the major peak of protease activity involving fractions 18–42 obtained in the last chromatographic step. After concentrating these fractions, SDS-PAGE was done (Fig. 2a) where it showed a prominent band at 35 kDa (Fig. 2b). The previous version of SDS-PAGE gel is presented in Supplementary Fig. S1.

**Table 1:** Data of the purification stages for UcB5 protease.

Purification step	Total activity (U)	Total protein content (mg)	Specific activity (U/mg)	Purification fold (*x*-fold)	Recovery (%)
Initial supernatant	621,427.0	1412.6	439.9	1.0	100
(NH_4_)_2_SO_4_ fractionation	579,791.4	627.6	923.8	2.1	93.3
Phenyl-Sepharose 6FF	276,535.0	56.1	4929.3	11.2	44.5
DEAE-Sepharose CL-6B	166,542.4	30.5	5460.4	12.4	26.8
Sephadex G-75	106,264.0	18.3	5806.8	13.2	17.1

### Biochemical properties of UcB5

**Figure 3:**
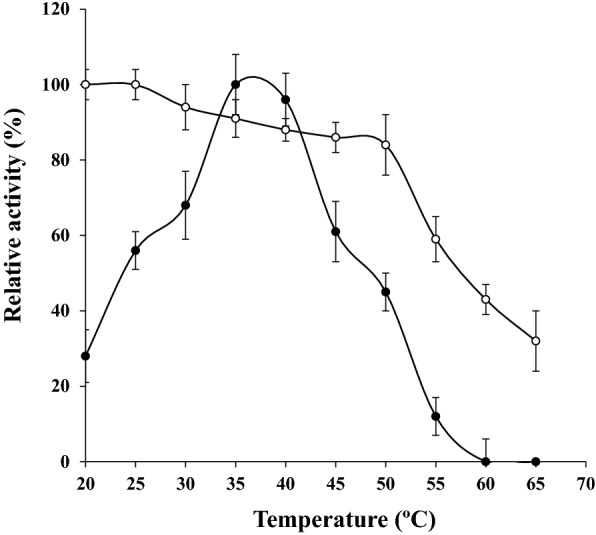
Impact of the temperature on both the proteolytic activity (-●-) and the stability (-○-). The temperature impact on the enzymatic activity was tested at pH 7.0 with 0.2 M Tris–HCl buffer using the substrate azocasein. The temperature stability of the UcB5 was studied by its incubation without substrate in 0.2 M Tris–HCl (pH 7.0) for 60 min. By the end of the incubation, the remaining activity was assessed. There is a highly positive correlation between the enzyme activity and its thermal stability since the correlation coefficient *r* = 0.738**. The two-tailed *P-*value is given as 0.015, this yields a highly significant linear correlation. While there is a highly negative correlation between the enzyme stability and temperature cleared from the correlation coefficient *r* = − 0.915***. The two-tailed *P-*value is given as 0.000, this yields a very highly significant linear correlation. The estimated regression equation can be expressed as enzyme stability = 139.9–1.47 temperature.

**Figure 4:**
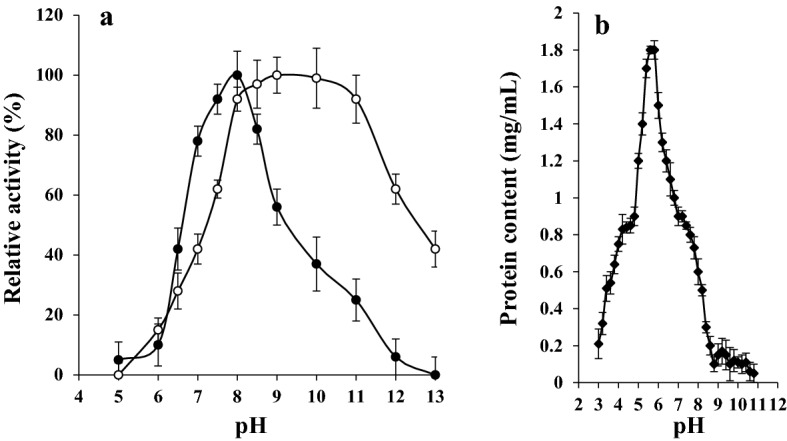
Impact of pH on both the proteolytic activity (-●-) and the stability (-○-) (**a**). The reaction temperature was adjusted to 35 °C in the presence of azocasein. The pH stability of the pure enzyme was inspected by its incubation without the substrate at 35 °C for 60 min with various pHs, then the remaining enzymatic activity was assessed at a pH value of 8.0. The isoelectric pH based on the protein precipitation pattern is shown in (**b**). There is a weak positive correlation between the enzyme activity and its pH stability since the correlation coefficient *r* = 0.487. The two-tailed *P-*value is given as 0.109. There is a weak negative correlation between the pH and the precipitated protein since the correlation coefficient *r* = *− *556***. The two-tailed *P-*value is given as 0.000 and the estimated regression equation can be expressed as protein content = 1.522–0.122 pH.

The best reacting temperature for the proteolytic activity of UcB5 against the substrate azocasein was detected at 35 °C (Fig. 3). In addition, the enzyme was thermally stable below 55 °C for 60 min. It retained 84% of its initial activity at 50 °C. The ideal pH for the proteolytic activity of UcB5 against the substrate was established at pH 8.0 (Fig. 4a). Furthermore, the pH stability of UcB5 was found at 8.0 to 11.0 for 60 min (Fig. 4a). At pH 8.0–11.0 the enzyme retained 92% of its original activity. As reflected by protein precipitation, the isoelectric point for the protein structure of this enzyme was found at pH 5.6 ± 0.2. The precipitation level reached 1.8 mg protein/mL (Fig. 4b).

### Amidolytic activity and kinetic parameters using synthetic substrates

Our results indicated that the enzyme has shown different degrees of amidolytic activities against the tested chromogenic substrates (Table 2). The standard protease substrate for chymotrypsin and subtilisin proteases, N-Succ-Ala-Ala-Pro-Phe-pNA was the furthermost substrate degraded by the UcB5 showing an amidolytic activity of 28.9 µmol min^−1^ L^−1^. Also, the UcB5 has degraded the chromogenic substrate D-Val-Leu-Lys-pNA, and D-Phe-Pip-Arg-pNA. However, it has shown the lowest action against D-Val-Leu-Arg-pNA. The kinetic parameters for the UcB5 against N-Succ-Ala-Ala-Pro-Phe-pNA substrate were; *K*_m_ of 0.16 mM and *K*_cat_/*K*_m_ of 301 mM^−1^ S^−1^.

**Table 2:** Amidolytic activity of the UcB5 on the chromogenic substrates.

Chromogenic substrate	Peptide sequence	*K*_m_ (mM)	*K*_cat_ (S^−1^)	*K*_cat_/*K*_m_ (mM^−1^ S^−1^)	Amidolytic activity (µmol^−1^ min^−1^ L^−1^)
S7388	N-Succ-Ala-Ala-Pro-Phe-pNA	0.16	48.15	300.94	28.86
V7127	D-Val-Leu-Lys-pNA	0.58	26.92	46.41	1.09
V6258	D-Val-Leu-Arg–pNA	39.53	4.08	0.10	0.02
P7021	D-Phe-Pip-Arg-pNA	0.37	8.19	22.13	0.04

Five microliters of the enzyme in 20 mM Tris–HCl buffer at pH 8.0 was incubated per 100 µL of a synthetic substrate at 0.02–0.15 mM concentrations at 37 °C. The colored para nitroaniline liberated from the reaction was measured at *A*_405_ nm. Depending on the velocity of the reaction, the kinetic parameters were deduced from the Lineweaver–Burk plot.

### Inhibition study for UcB5

The study of the effect of protease reagents and cations on enzyme activity (Table 3) offers an initial understanding of the nature of the tested enzyme, the nature of the active site, and the cofactor supplies. During the investigation of the impact of cations on the amidolytic activity, it was found that none of them was an activator for the enzyme. The amidolytic activity in absence of metals was considered as 100%, therefore, the relative activity was 92% for Ba^2+^, 87% for Co^2+^, 62% for Zn^2+^, 102% for Fe^3+^, 84% for Ca^2+^, 98% for Mg^2+^, 65% for Cu^2+^, 49% for Mn^2+^, 67% for Cd^2+^, 103% for Ag^2+^, and 47% for Hg^2+^ ions.

**Table 3:** Impact of the protease inhibitors on the proteolytic activity of UcB5.

Protease reagent	Concentration (mM)	Relative activity (%)
Control	NA	100 ± 1.2***
SBTI^a^	0.2	31.1 ± 3.1**
	0.5	0.0 ± 0.1
TLCK^b^	0.1	1.2 ± 0.0
PMSF^c^	2.0	17.3 ± 1.0***
	5.0	0.0 ± 0.1
EDTA^d^	10	100.1 ± 5.1***
DMSO^e^	10	105.2 ± 4.2***
EGTA^f^	10	100.2 ± 2.5***
DTT^g^	10	100.5 ± 1.8***
	50	99.8 ± 6.5***
EAM^h^	20	102.1 ± 4.0***
PHMB^i^	2	99.7 ± 8.0**
β-Mercaptoethanol	10	103.4 ± 6.4***
	50	98.9 ± 5.0***
Leupeptin	0.02	82.0 ± 3.5***
	0.05	78.5 ± 2.9***
Pepstatin A	0.02	15.7 ± 0.9***
	0.05	0.0 ± 0.0
Aprotinin	0.1	3.0 ± 0.1***
2,2′-Bipyridine	0.1	102.2 ± 4.2***
*o*-Phenanthroline	0.1	99.5 ± 3.3***

These reagents were incubated with 1.0 × 10^–4^ mg of the chromogenic substrate S7388 and 2.0 × 10^–3^ mg of UcB5 in 100 µl of 20 mM Tris–HCl buffer (pH 8.0) for 3 min at 37 °C. The released pNA was quantified at *A*_405_ nm and the proteolytic activity in the absence of reagents was considered 100% activity.

^a^Soybean trypsin inhibitor; ^b^tosyl-lysyl-chloromethylketose; ^c^phenylmethylsulfonylfluoride; ^d^ethylenediaminetetraacetic acid; ^e^dimethyl sulfoxide; ^f^ethylene glycol-O,O′-bis-[2-amino-ethyl]-N, N, N′, N′-tetraacetic acid; ^g^1,4-dithiothreitol; ^h^aminobenzamidine ethyl acetimidate; ^i^p-hydroxymercuribenzoate. There was a statistically significant difference among the protease reagents as determined by one-way analysis of variance (ANOVA) (F calculated = 369.69, F tabled = 2.4, P = 0.000). *P significant at P < 0.05; **P highly significant at P < 0.01; ***P very highly significant at P < 0.001; Student’s one-sample t-test.

Concerning the impact of protease reagents, it was found that the protease activity of UcB5 was inhibited by TLCK (1.2% relative activity), PMSF (17.3% relative activity), SBTI (31.3% relative activity), aprotinin (3.0% relative activity), pepstatin A (15.7% relative activity), and leupeptin (78.5% relative activity) but was not influenced by 2,2′-bipyridine (102.2% relative activity), DTT (100.5% relative activity), *o*-phenanthroline (99.5% relative activity), β-mercaptoethanol (98.9% relative activity), and the two metalloprotease inhibitors EDTA (100.1% relative activity) and EGTA (100.2% relative activity) (Table 2). Furthermore, our results indicated that the mercaptide-forming agents PHMB (99.7% relative activity) and EAM (102.1% relative activity) didn’t influence the proteolytic activity.

### Substrate specificity of UcB5

This was determined against several natural proteins at 0.5% (w/v) concentration. Considering the activity of the UcB5 against casein was 100%, the relative activities against fibrin, gelatin, mucin, hemoglobin, fibrinogen, elastin, collagen, IgG, and serum albumin were 32.0, 65.6, 6.8, 23.8, 76.3, 11.2, 42.5, 0.0, and 12.7%, respectively. The proteolytic action against plasma proteins was also confirmed during the next experiment during testing its anticoagulant activity (Supplementary Table S1). In the presence of UcB5, the clotting time of blood serum was extended to 81 s constituting 3.5-fold than the clotting time without enzyme.

### Cell damaging activity

During the in vitro experiment presented in Supplementary Table S1, UcB5 at a dose of 15 µg enzyme/ml has shown 63.8% cell death of the cultured HT29 cell line. Besides, it elicited a 3.9-fold rise in the hemolysis of RBCs. Furthermore, a comparative in vivo study with an active and a heat-inactivated protease preparation was performed in an attempt to determine whether there is a relationship between its toxicity and enzymatic activity. The active preparations were employed at 60 µg to 4 µg protease/mouse body weight (Supplementary Table S2). At concentrations of 60 µg and 30 µg, death has occurred for all tested animals within 2 h, whereas 4 and 1 out of six mice have died within 48 h with 15 µg and 8 µg treatments, respectively. No lethality was noted with 4 µg treatment of the enzyme. On the other hand, none of the mice were killed by the heat-inactivated enzyme preparation. The calculated LD_50_ at 48 h was 15.75 µg protease/mouse body weight (Supplementary Table S2).

**Figure 5:**
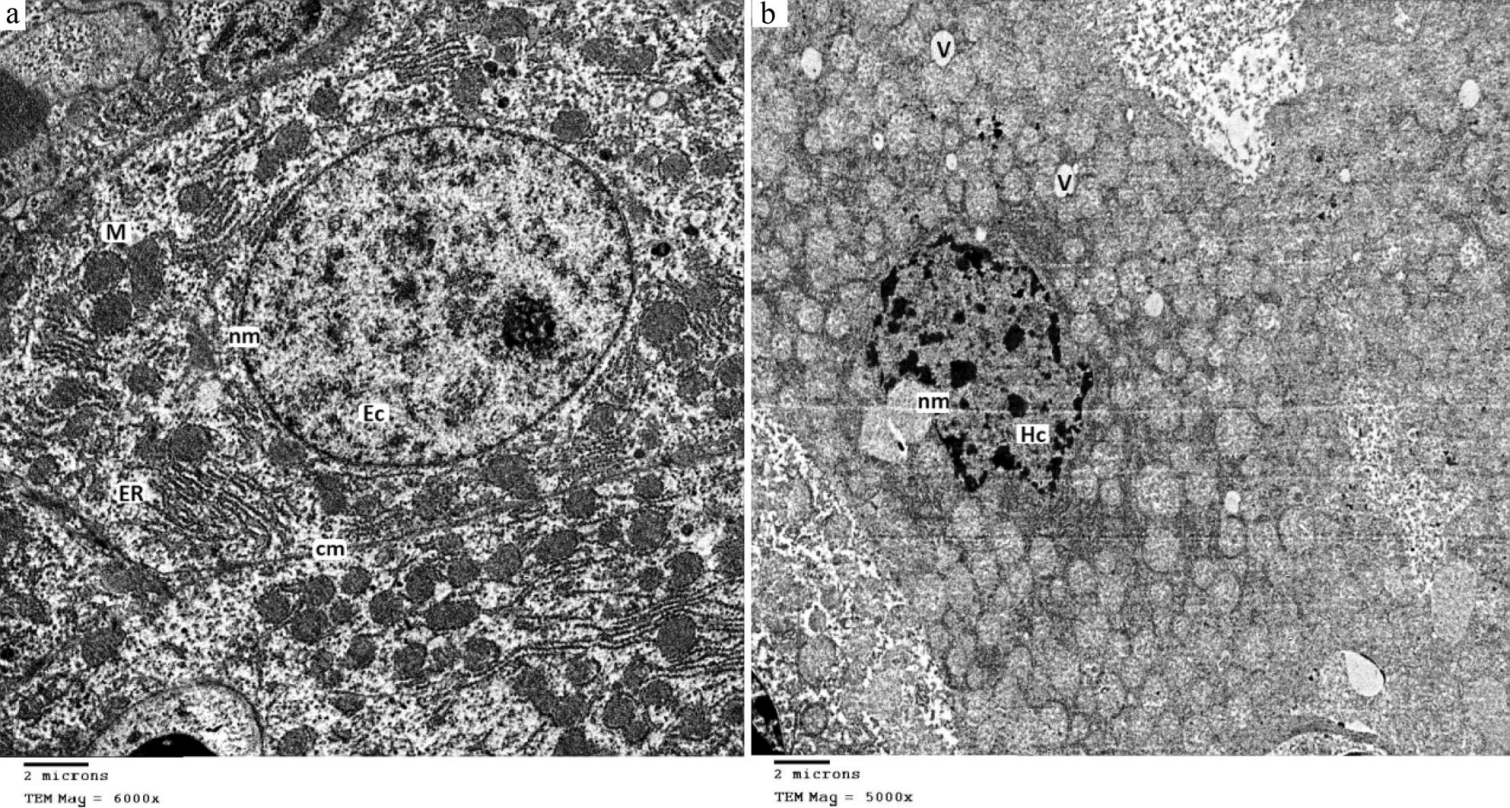
TEM micrographs showing the ultrastructural changes in the hepatocytes of treated and control mice. (**a**) A typical hepatocyte shows an intact cell membrane (cm) and intact nuclear membrane (nm) containing euchromatin (Ec). Also, it shows an intact rough endoplasmic reticulum (ER) and extensive normal mitochondria (M). (**b**) A liver cell of a mouse that has received an active preparation of UcB5 protease. It shows vesiculation (V) and malformed nuclear membrane (nm) with heterochromatin (Hc). It shows the disappearance of the cell membrane and rough endoplasmic reticulum. The in vivo cell damaging activity was evaluated by experimenting with the BALB/c mice housed in six groups. Briefly, a purified UcB5 in a total of 1 ml volume was inoculated in every mouse by the intraperitoneal route. Every mouse was inoculated with a heat-inactivated enzyme preparation for the universal control group. Livers were removed from the sacrificed and the control animals and fixed. Finally, ultrathin sections of 70 nm thickness were taken with RMC ultramicrotome and supported on copper grids for electron microscopic examination under JEOL 1010 TEM.

The TEM study on the affected mice revealed that UcB5 has induced vesiculation (V) inside the hepatocytes and the nuclear membrane (nm) became malformed with the accumulation of heterochromatin (Hc). The cell membrane and the rough endoplasmic reticulum (ER) of hepatocytes completely disappeared (Fig. 5).

## Discussion

The ability of pathogenic microorganisms to cause diseases depends on the development of extracellular proteases, according to several published research. Although the precise method of action is unclear, it appears that these microbial enzymes interfere with the protease system of host^17^. In our former studies, we reported the pathogenesis of the KB76 protease of *Brevibacterium otitidis*^18^ and the ZuhP13 protease of *Pseudomonas aeruginosa*^17^. In this paper, we have separated and characterized a possible cytotoxic enzyme nominated as UcB5 protease from the bacterium *S. typhimurium*.

The most effective protease-producing strain, strain UcB5, was identified by the *16S rDNA* gene sequencing data as *S. typhimurium*. After 48 h of incubation, the UcB5 protease production (125 U/mL) and the bacterial growth were synchronized at the highest level (Fig. 1). Thus, it can be inferred that UcB5 protease, like ZuhP13 protease of *P. aeruginosa*, is a main enzyme required for bacterial development^17^. Contrary to the protease of the fish pathogen, *Yersinia ruckeri* has demonstrated the best rank of productivity at 12 h^19^. Interestingly, the UcB5 was produced in a basal medium devoid of the substate casein (see materials and methods) which is an indication of a constitutive, not an inducible enzyme.

The final specific activity of the isolated enzyme increased by 13.2-fold, with a 17.1% recovery. SDS-PAGE of the last column proteins (Fig. 2a) revealed a distinct band at 35 kDa (Fig. 2b). This molecular mass coincides with the pathological proteases of *P. aeruginosa*^20^ while, unlike the proteases of *Legionella pneumophila* (38 kDa^21^), *Vibrio pelagius* (39 kDa^22^) *Vibrio parahaemolyticus* (43 kDa^23^), *Br. otitidis* (47 kDa^18^), and *Y. ruckeri* (47 kDa^19^).

With a resemblance to the virulent protease of *Y. ruckeri*^19^, 35 °C was shown to be the optimal reaction temperature for the proteolytic activity of UcB5 (Fig. 3). While 40 °C was best for the ZuhP13 protease^17^, and 50 °C was best for the ME4 protease^24^. Furthermore, UcB5 was found to be heat-stable below 55 °C for 60 min (Fig. 3). Referring to the literature, it is more heat-stable in evaluation with *Y. ruckeri* virulent protease (complete inhibition at 55 °C^19^) and more temperature-labile in comparison with san-ai protease of *P. aeruginosa* (stable at 60 °C for 90 min^25^.

The proteolytic activity of UcB5 was shown to be best at pH 8.0 (Fig. 4a) which is similar to the pathological proteases of *Y. ruckeri*^19^, *Bacillus cereus* strain BG1^26^, and strain KCTC 3674^27^. The major decline in the proteolytic activity at lower pH values may be due to that, the lower pH values coincide with the isoelectric point (p*I*, 5.6 ± 0.2, Fig. 4b). The KB76 protease of *Br. otitidis*^18^ and the corneal-damaging protease of a strain of *P. aeruginosa*^4^ were reported to have similar p*I*s by other investigators as well. Additionally, the pH stability of UcB5 protease was found between 8 and 11 for 60 min (Fig. 4a) similar to the protease of *Aeromonas veronii* PG01^28^ as opposed to the ZuhP13 protease^17^ and the ME4 protease of *P. aeruginosa*^24^ which were stable at pH 6–9 for 60 min.

Against the investigated chromogenic substrates, the enzyme displayed varying degrees of amidolytic activity (Table 2). The standard protease substrate for chymotrypsin and subtilisin proteases, N-Succ-Ala-Ala-Pro-Phe-pNA was the furthermost substrate degraded by the UcB5 showing an amidolytic activity of 28.9 µmol min^−1^ L^−1^. Moreover, the UcB5 degraded the chromogenic substrate D-Val-Leu-Lys-pNA which is the standard substrate of plasmin proteases, and D-Phe-Pip-Arg-pNA which is the substrate of thrombin proteases. However, the enzyme demonstrated the least activity against D-Val-Leu-Arg-pNA, the chromogenic substrate of kallikrein protease types. From these findings, we can conclude that UcB5 is similar to subtilisin or chymotrypsin because it hydrolyzed Lys-peptide bonds more readily than Arg-peptide bonds. Interestingly, subtilisin UcB5 attacked D-Phe-Pip-Arg-pNA more effectively than D-Val-Leu-Arg-pNA like the subtilisin FS33 protease^29^ which set it apart from other subtilisins.

The kinetic values for UcB5 against the substrate N-Succ-Ala-Ala-Pro-Phe-pNA were; *K*_m_ of 0.16 mM and *K*_cat_/*K*_m_ of 301 mM^−1^ S^−1^. Regarding other subtilisins, ZuhP13 from *P. aeruginosa* showed a catalytic efficacy of 4.62 × 10^7^ M^−1^ S^−1^ with *K*_cat_ of 1.27 S^−1^^17^. The catalytic efficacy of ZapA from *Proteus mirabilis* N17-12 against Phe-Ser was 291 mM^−1^ S^−1^, *K*_m_ was 13.6 µM, and *K*_cat_ was 3.96 S^−1^ whereas, the catalytic efficacy against Phe-Leu was 13 mM^−1^ S^−1^, *K*_m_ was 2.3 µM, and *K*_cat_ was 0.03 S^−1^^30^.

During the examination of the effect of cations on amidolytic activity, none of the cations was an enzyme activator. When considering the effects of protease reagents, it was found that 2,2'-bipyridine, DTT, *o*-phenanthroline, β-mercaptoethanol, and the two metalloprotease inhibitors (EDTA and EGTA) didn't affect the activity of UcB5 (Table 2). Furthermore, the mercaptide-forming agents (PHMB and EAM) didn't affect the proteolytic activity. Accordingly, we propose that tryptophan (indole) and serine (hydroxy) groups are sited at or close to the active center of UcB5. Combining our findings, we propose that, UcB5 belongs to serine proteases similar to the *E. coli* espP virulent protease^31^. Referring to the literature, we noticed that virulent serine proteases are rare in comparison with virulent metalloproteases. The latter type was discovered in the extracts of *Y. ruckeri*^19^, *P. mirabilis* N17-12^30^, and almost all strains of *P. aeruginosa*^24,25^.

The relative activity of UcB5 against fibrin, gelatin, mucin, hemoglobin, fibrinogen, elastin, collagen, IgG, and serum albumin were 32.0, 65.6, 6.8, 23.8, 76.3, 11.2, 42.5, 0.0 and 12.7%, respectively. The unproteolysis of the immunoglobulin type G may be promising in the production of chimeric proteins made from UcB5 and IgG to target specific unwanted cells. The subsequent experiment, which evaluated the anticoagulant properties of enzyme, further validated the proteolytic action against plasma proteins (Supplementary Table S1). In the presence of UcB5, the clotting time of blood was extended to 81 s constituting 3.5-fold than the clotting time without enzyme. The capability of UcB5 to destroy fibrin and fibrinogen supports the idea that microbial proteases facilitate bacterial translocation inside the body. As a result, we are in favor of utilizing a suitable protease inhibitor in conjunction with antibiotics rather than just antibiotics alone to treat bacterial infections inside the human body^32^.

During the in vitro experiment described in Supplementary Table S1, UcB5 has shown 63.8% cell death of the cultured HT29 cell line. Besides, it elicited a 3.9-fold rise in the hemolysis of RBCs. As a result, the disintegration of the cell and RBC membrane appears to be a phase in the mode of action of UcB5. When this happened, the enzyme began to react with hemoglobin and other important internal proteins. This characteristic of the UcB5 might be the cause of hemorrhage that was observed in the internal cavities of the thorax and the abdomen of the dissected animals. The presented hemolytic and hemorrhagic activities of the UcB5 coincide with the protease A of *V. parahemolyticus* no. 93^23^ and ZuhP13 protease of *P. aeruginosa*^17^.

Upon injection of UcB5 in mice, vesiculation (V) occurred inside the hepatocytes and the nuclear membranes (nm) became deformed due to the buildup of heterochromatin (Hc). In addition, TEM revealed that the cell membrane and the rough endoplasmic reticulum (ER) of hepatocytes were totally vanished (Fig. 5). The cellular and nuclear lysis caused by UcB5 may be due to necrosis, not apoptosis since there is no impairment to the mitochondrial configurations (M). Concerning cited pathological proteases, *Pseudoalteromonas* sp. N10 and *V. vulnificus* proteases generated an impairment of the muscle proteins and exhibited deep wound necrosis, respectively^33^. Fish experienced significant cellular damage due to *Y. ruckeri* protease^19^. On the other hand, researchers have demonstrated that proteases of streptococci induced both necrotizing fasciitis and cellular apoptosis of host tissues^34^.

## Supplementary Information

Supplementary Information.

## Data Availability

All data generated or analysed during this study are included in this published article and its supplementary information files.

## Abbreviations

DEAE: Diethylaminoethyl
DMSO: Dimethyl sulfoxide
DTT: 1,4-Dithiothreitol
EAM: Aminobenzamidine ethyl acetimidate
EDTA: Ethylenediaminetetraacetic acid
EGTA: Ethyleneglycotetraacetic acid
IgG: Immunoglobulin class G
LB broth: Luria–Bertani broth
MTT: 3-(4,5-Dimethylthiazol-2-yl)-2,5-diphenyltetrazolium bromide reagent
PHMB: Polyhexamethylene biguanide
p*I*: Isoelectric point
PMSF: Phenylmethylsulfonyl fluoride
pNA: P-nitroaniline
SBTI: Soybean trypsin inhibitor
SDS-PAGE: Sodium dodecyl sulfate–polyacrylamide gel electrophoresis
SS agar: *Salmonella Shigella* agar
TEM: Transmission electron microscope
TLCK: Tosyl-l-lysine chloromethyl ketone
