# Supplementary material for: A putative cytotoxic serine protease from Salmonella typhimurium UcB5 recovered from undercooked burger
Source: Sci Rep. 2023 Mar 9;13:3926. doi: 10.1038/s41598-023-29847-8 (PMC9998444; doi:10.1038/s41598-023-29847-8)
Supplement: Supplementary file 1 — Supplementary Information. [file 41598_2023_29847_MOESM1_ESM.docx]

**Supporrting information**

**Supplementary Table S1** *The in vitro* proteolytic action of UcB5 protease

| *In vitro* action | Treated | Blank |
| --- | --- | --- |
| Cytotoxicity *^a^* | 63.8±3.5 | 2.3±0.1 |
| Hemolysis *^b^* | 3.5±0.2 | 0.9±0.1 |
| Anticoagulant activity *^c^* | 81.0±3.5 | 23.0±2.4 |

*^a^* Percent of death of HT29 cells after the incubation for 24 h with 15.0 µg UcB5/mL solution. This assay was done by the MTT method [16]. For negative controls, physiological saline was used instead of the active protease preparations while, for blanks, a medium without cells was used. *^b^* Percent of cell-damaging activity against RBCs due to the purified enzyme. This assay was done by vortexing identical volume sizes of 15 µg UcB5 protease/mL and 4% (v/v) RBCs suspension. Incubation was done at 37°C for 90 min then the quantity of liberated hemoglobin was assessed colorimetrically. The degree of absolute hemolysis was done by mixing RBCs suspension with 1% (v/v) triton X-100. *^c^* Time of clotting in seconds for the blood plasma. This was done by mixing 100 µL of blood serum with equal volumes of each thromboplastin and kaolin. After 2 min at 37°C in a water bath, 100 µL of 0.3% CaCl_2_ and 0.1 mL of the purified enzyme at 15 µg/mL concentration was added. The clotting time in the presence of enzyme was then determined in comparison with blanks containing an equivalent amount of the physiological saline instead of the purified enzyme. For each case of in vitro damage, the independent samples t-test were performed to study if the difference between the treated and the control samples were significant or not. Levene's test for equality of variances is also done and the resulted P-value indicated that equal variances are assumed since P = 0.12, 0.422, and 0.632, respectively. All are higher than 0.05. The results of the t-test for each case assure that the difference is significant and doesn’t return to chance since the significant P-value for all cases equals zero.

**Supplementary Table S2** LD_50_ calculation for the UcB5 protease using Karber’s method

| Product  = a*b | Mean  = b | Dead animal | Dose difference (µg/mouse body weight)  = a | Dose (µg/mouse body weight) |
| --- | --- | --- | --- | --- |
| 0 | - | 6 | 0 | 60 |
| 180 | 6 | 6 | 30 | 30 |
| 75 | 5 | 4 | 15 | 15 |
| 17.5 | 2.5 | 1 | 7 | 8 |
| 2 | 0.5 | 0 | 4 | 4 |
| T*^a^*= 274.5  n*^b^*= 6 |  |  |  |  |

*^a^* total product

*^b^* number of tested animals in each group


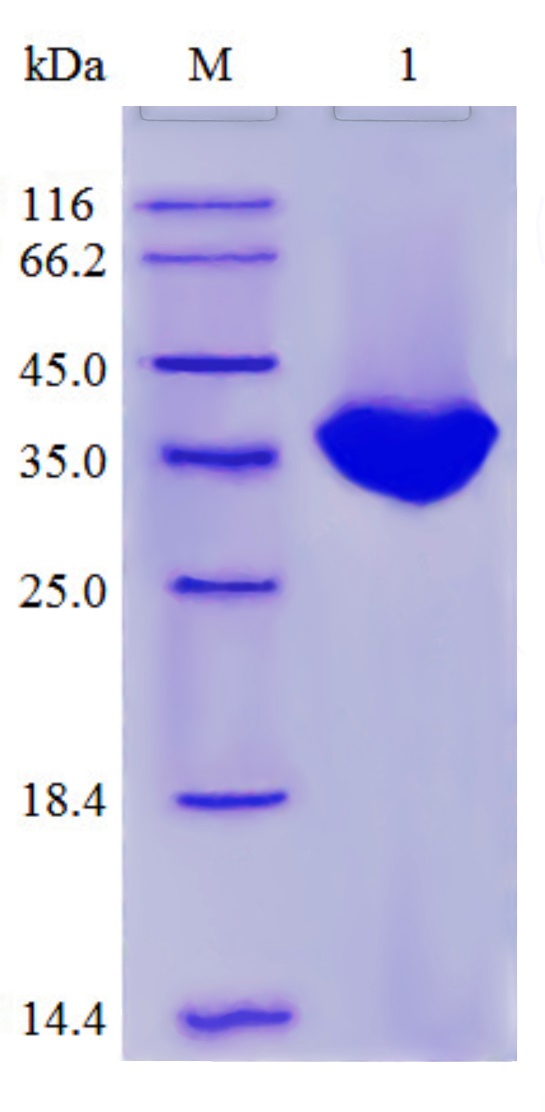


**Supplementary Figure S1** SDS-PAGE analysis using 5% (w/v) stacking gel and 15% (w/v) separating gel.
